# Understanding Laboratory Testing for SARS-CoV-2

**DOI:** 10.3390/children8050355

**Published:** 2021-04-29

**Authors:** Ritu Cheema, Dean A. Blumberg

**Affiliations:** Department of Pediatrics, Division of Pediatric Infectious Disease, University of California, Davis, School of Medicine, Sacramento, CA 95817, USA; dablumberg@ucdavis.edu

**Keywords:** SARS-CoV-2, COVID, pandemic, serology, nucleic acid amplification test, RT-PCR, rapid antigen test, antibody testing, point-of-care test

## Abstract

The SARS-CoV-2 pandemic has impacted millions of lives worldwide. Molecular assays and serological tests have been approved under EUA (emergency use authorization) by the FDA (food and drug administration), given these unprecedented times. These tests are not only critical for confirming the clinical diagnosis and making therapeutic decisions but also play an important role in the understanding of the epidemiology of the pandemic. There is limited experience with currently available tests and differences may exist among tests even using similar technology. The focus of this review is to improve clinicians’ understanding of SARS-CoV-2 test procedures including their limitations. We discuss the impact of different host and environmental factors on test results.

## 1. Introduction

About 130 million COVID-19 (Coronavirus disease 2019) cases have been confirmed worldwide as of 4 April 2021 [1] although the actual number of cases is likely significantly higher. On 4 February 2020, in the US, the Secretary of Health and Human Services determined that circumstances justified the authorization of emergency use of in vitro diagnostics for detection of the novel coronavirus (SARS-CoV-2) due to a public health emergency [2]. On 29 February 2020, the FDA issued specific guidance approving several commercial assays, expanding the testing abilities for COVID-19. Since clinical specimens were not easily available during the early months of pandemic, the analytical and clinical performance of these tests was derived from gene specific RNA, synthetic RNA or whole genome viral RNA. More recently, the FDA has developed a reference panel from live virus which allows a direct comparison of sensitivity across these tests. These tests are critical for confirming clinical diagnosis, assisting with therapeutic decision making as well as understanding the epidemiology of the pandemic. They help with timely isolation to help curb the pandemic, or mitigation strategies and for the appropriate allocation of personal protective equipment (PPE), especially when in short supply. There is limited experience with multiple currently available tests and result differences may exist among tests even using similar technology. The focus of this review is to improve clinicians’ understanding of SARS-CoV-2 test procedures including their limitations and interpretation.

## 2. Tests for SARS-CoV-2 Infection

Broadly, there are two categories of tests available for SARS-CoV-2 infection: diagnostic and serologic. The first category identifies acute infection by detection of viral nucleic acid or viral antigens. The most common nucleic acid amplification tests (NAAT) also called molecular tests involve reverse transcription of the viral RNA followed by nucleic acid amplifications (RT-PCR). Viral antigen detection identifies viral structural proteins of the virus. The second testing category detects the immune response to viral infection and thus is used to identify previous infection. Serologic tests which detect IgM and/or IgG antibodies to the SARS-CoV-2 and are not used to diagnose a current infection.

**Molecular assays:** Various techniques for nucleic acid amplification include reverse transcription polymerase chain reaction (RT-PCR—requires thermal cycling), isothermal amplification (does not require thermal cycling), CRISPR-based assays (clustered regularly interspaced short palindromic repeats), SHERLOCK (Specific High Sensitivity Enzymatic Reporter UnLOCKing) and next-generation sequencing. RT-PCR has been the most common method used for detection of viral nucleic acid and will be the focus herein [3,4,5]. The timing of test results varies from 15–30 min (point-of-care) to up to 3–4 h (laboratory-processed). Delayed reporting may be attributed to factors including collection, transport, data analysis. Point-of-care (POC) molecular assays may be more useful in settings like emergency departments and urgent care facilities where results are needed quickly. Since the FDA has approved POC tests under an EUA, the clinical accuracy of these tests is being closely monitored and, hence, updated FDA alerts on these tests should be regularly monitored by clinicians.

During the initial part of the pandemic, tests were performed by the Centers for Disease Control and Prevention (CDC) and local public health departments. Very shortly, various commercial reference laboratories and hospital laboratories developed their own tests. The tests are primarily performed on nasopharyngeal swabs, nasal swabs, and saliva (upper respiratory specimens) but can also be performed on lower respiratory tract samples. COVID-19 is an enveloped RNA virus and hence real time reverse (RT-PCR) tests designed by different manufacturers target the presence of one or several SARS–CoV-2-specific genes. Amplification targets include nucleocapsid (N1, N2, N3), envelope (E), spike (S) and the RNA dependent RNA polymerase (RdRp) genes and utilizes human ribonuclease P as an extraction control [6,7]. Each sample source requires its own validation and optimization. Typically, a probe strategy is used to detect amplification of the template. This requires addition of a short probe, complementary to the amplified sequence, that contains a fluorescent molecule and a quencher molecule. The latter is released due to probe degradation upon subsequent amplification cycles, thus freeing the fluorescent molecule for detection. Fluorescence increases as copies of the virus DNA are made. If the fluorescence level crosses a certain threshold, the test is positive. In certain situations, results may be discordant indicating identification of only one of two or more gene targets and can be considered presumptively positive due to high specificity of these assays. Testing could be repeated as well if the patient is early in the disease course. All clinical samples should exhibit fluorescence growth curves in the ribonuclease P (RNase P) reaction that cross the threshold line within 40.00 cycles, thus indicating the presence of the human RNase P gene. Several factors can result in failure to detect RNase P in clinical specimens like reagent or equipment malfunction, improper assay set up and execution, poor collection or loss of specimen integrity resulting in absence of sufficient human cellular material, improper extraction of nucleic acid from clinical materials, thus resulting in loss of RNA and/or RNA degradation. This test can take a total of up to 4 h including RNA extraction from the sample [7].

Currently all authorized tests are qualitative (providing a result that is positive, negative, or indeterminate) rather than quantitative (providing a quantitative assessment or titer). It is also important to understand that each test has its own limit of detection, defined as lowest possible concentration of viral RNA that can be detected under the experimental conditions in at least 95% of all reactions. Hence, a lower limit of detection (LoD) indicates higher sensitivity but would emphasize that LoD is for the specimen tested, not for the purified RNA extract produced at sample processing. The LoD of the CDC’s 2019-nCoV Real-Time RT-PCR Diagnostic Panel was found to be 10^0^ copies/μL which would be same as 1 copy/μL [7]. It is important to remember that various tests may use different viral gene targets for detection and, hence, vary in their performance due to difference in their limits of detection, especially when lower RNA concentrations are present in the specimen. NAATs have high analytic sensitivity in ideal settings indicating the ability to accurately detect even low levels of viral RNA but clinical performance can be variable based on multiple factors including specimen quality, duration of illness, or specific assay used.

Overall, the sensitivity (proportion of subjects with disease in whom the test is positive) and specificity (proportion of subjects without disease in whom the test is negative) of molecular tests is reported generally high. The analytical sensitivity (lowest amount of target in a sample that can be measured accurately by that test, also known as limit of detection) of different assays is available at the in vitro-diagnostic EUA site [2]. It is worth mentioning that whenever a new test is evaluated by comparing to a non-reference standard, it may not be entirely possible to calculate estimates of sensitivity, specificity, positive predictive value, negative predictive value, and the positive and negative likelihood ratios without bias. Hence, it is recommended instead to use positive percent agreement and negative percent agreement with the non-reference standard preferred for description of their results. The CDC’s 2019-nCoV Real-Time RT-PCR Diagnostic Panel (using N1 and N2 assay) (which was the first test introduced during the pandemic for clinical testing) has positive percent agreement (the proportion of non-reference standard positive subjects in whom the new test is positive) of 100% and a negative percent agreement (the proportion of non-reference standard negative subjects in whom the new test is negative) of 100% when confirmed with genetic sequencing and/or virus culture [7]. Lierberman et al. compared the analytical performance of laboratory-developed assay at their local laboratory, based on CDC primer sets and our commercially available assays for SARS-CoV-2 (Cepheid, DiaSorin, Hologic Panther, and Roche Cobas) on nasopharyngeal swabs. A total of 100% agreement was noted across specimens between their CDC-based laboratory developed test (LDT) and Cepheid Xpert Xpress followed very closely by Hologic Panther Fusion, DiaSorin Simplexa and Roche Cobas 6800. All the tests were 100% specific, using CDC-based LDT as the gold standard [8].

A systematic review of rapid POC molecular tests earlier during the pandemic using confirmed SARS-CoV-2 samples reported sensitivity ranging from 68 to 100% with the average at 95.2% and specificity 98.9% based on 13 evaluations in 11 studies on 2255 samples [9]. It is worth mentioning that certain POC tests with results reported within 5 min like the Abbott ID Now assay were developed using isothermal nucleic acid amplification of the RNA polymerase viral target but have been found to have lower sensitivity especially in samples with lower viral load [10].

Pooled sample testing has generated interest recently with the purpose of enhancing the capacity of laboratories for testing. This involves testing pooled samples generated by mixing several samples together and then retesting individual specimens only if the pooled test is positive [11]. Although this strategy aims to utilize the same amount of reagent for testing multiple individuals at the same time, there is a potential concern for false negative results due to dilution of samples, resulting in fewer viral copies. This approach is more useful in the settings with low prevalence of cases compared to high prevalence where repeated testing of positive pools could delay the detection of true positive individuals.

**Viral antigen tests:** A rapid antigen test has been the latest test to join the market after FDA approval and provides results in 15–30 min as it does not require extraction and amplification steps. This test is typically performed on nasopharyngeal or nasal swabs and utilizes the lateral flow technique to detect specific viral proteins. If the target antigen is present in sufficient concentrations, it will bind to specific antibodies, which are fixed to a paper strip enclosed in a testing case. A detectable signal can be easily read by eye. The antigen detected is expressed only when the virus is actively replicating; therefore, such tests are best used to identify acute or early infection. Although these tests have the advantage of rapid detection and high positive predictive value, they may yield false negatives if the viral protein production is low. The antigen level in specimens collected either before symptom onset, or late in the course of infection, may be below the limit of detection of the test. A wide range of sensitivity of 0 to 94 percent with average of 56% for rapid antigen tests was noted in a review of 8 evaluations from 5 studies on 943 samples showed a specificity of 99%. Of note, the average sensitivity increased to 93% if the specimen contained higher viral load [9]. Hence, a negative test may need to be confirmed by sensitive PCR test if clinical suspicion remains high. The performance of BinaxNOW (rapid antigen test detecting nucleocapsid protein antigen) was studied on both symptomatic and asymptomatic 868 subjects. This test had sensitivity of 93.3% at a cycle threshold (Ct) count of 30 and below (corresponded to a viral RNA copy number of approximately 1.9 × 10^4^ in that assay) but sensitivity decreased to 57.7%, when measured without a Ct threshold. Of note, the specificity remained at 99.9% with or without Ct value [12]. In a situation with a high index of suspicion for infection but negative rapid antigen test, either RT-PCR should be obtained, or a rapid antigen test can be repeated test at a later time (preferably within 2 days) to ensure infected individuals are not missed. Rapid antigen tests would also be useful where serial screening can be performed, for example in congregate settings and in outbreak scenarios where the individuals would need to be quickly identified for isolation purposes [13].

Of note, viral culture of specimens is not recommended at this time for biosafety concerns. In addition, current mRNA vaccine administration does not result in positive test results with NAATs or rapid antigen detection but may lead to a positive serology or antibody test.

## 3. Factors Influencing Interpretation of Results

Accuracy of these diagnostic tests will depend on timing of collection during the course of illness, quality of the specimen, and appropriate transport and processing. Being an RNA virus, SARS-CoV-2 has low stability and can be easily degraded by ubiquitous exogenous RNase enzymes and by RNases released at the time of cellular destruction.

**Sample collection:** Flocked swabs with an aluminum or plastic shaft are best to enhance the collection and release of cellular material. Swabs containing calcium alginate, wood, or cotton are not recommended as they may contain substances that inhibit PCR amplification. Swabs should be transferred into universal transport medium immediately after collection in order to preserve nucleic acid. Nasopharyngeal swabs appear to have the highest sensitivity, however other options include swabs from the oropharynx or anterior nares. The viral load is highest in lower respiratory tract compared to upper respiratory tract (e.g., viral load in nose/nasopharynx higher than oropharynx) [14]. It would mean that, for the same degree of sensitivity for a test, there is a higher chance of obtaining a positive result from sputum/bronchoalveolar samples compared to nasopharyngeal sample. However, it would not be practically feasible nor desirable for every suspected patient to undergo bronchoscopy for testing. Sputum induction is not recommended.

The cycle threshold (Ct) also known as quantification cycle (Cq) refers to the number of cycles in an RT-PCR assay needed to amplify viral RNA to reach a detectable level. The Ct value may be used as a rough indicator of copy number of viral RNA where specimens with lower cycle threshold values correspond to higher viral copy numbers. The cycle threshold of less than 40 has been defined as the cutoff for positivity by the FDA. The results cannot be compared across different tests as Ct values have not been standardized across different RT-PCR platforms. In a report involving 1070 collected specimens (bronchoalveolar lavage fluid, sputum, nasal swabs, fiberoptic bronchoscope brush biopsy, pharyngeal swabs, feces, and blood) from 205 patients with COVID-19, the mean cycle threshold values of all specimen types were more than 30 (<2.6 × 10^4^ copies/mL) except for nasal swabs with a mean cycle threshold value of 24.3 (1.4 × 10^6^ copies/mL), indicating higher viral loads in nasal swabs [15]. Live virus has also been detected in saliva by viral culture and, hence, saliva can be a promising non-invasive specimen for diagnosis, monitoring, and infection control in patients with this infection [16]. An average one-half log higher viral RNA levels in first morning saliva compared to nasopharyngeal samples acquired in the first 10 days of illness in a study of 70 patients hospitalized with COVID-19 [17]. Saliva testing causes less discomfort during collection, does not involve aerosol generation and could be a useful alternative in areas with shortage of personal protective equipment (PPE) and specimen sampling kits, both of which are needed during nasopharyngeal sample collection.

Home based testing also further decreases the need for PPE and is convenient for patients. In another study of 501 patients, nasal and mid-turbinate specimens self-collected by patients had sensitivity of 94% and 96% by RT-PCR, respectively, when using a nasopharyngeal sample collected by a health care worker as a comparator [18].

Viral RNA was detected in the blood of 6 of 57 patients and in anal swabs of 11 of 28 patients. Interestingly, all the patients with viremia and positive anal swabs had severe disease at the stage of their testing [19]. In another study of 59 infected patients, only 17.6% had gastrointestinal symptoms but viral RNA was detected in the stool samples from 48.1% of the patients while respiratory samples tested negative [20]. However, testing stool samples has not been validated in clinical laboratories in the US for either blood or stool samples.

**Sample storage:** Specimens should be stored at 2–8 °C for no more than 72 h after collection. Specimens should be quickly transported to laboratories as delays can affect the stability of specimens especially if not stored appropriately. If a delay is anticipated in testing or shipping, the specimen should be stored at <−70 °C.

**Laboratory factors:** Laboratory factors that may affect test accuracy include optimization of assay conditions (e.g., reagent conditions, incubation times, and temperatures), appropriate selection of controls (to ensure assay reliability and identification of experimental errors), efficient RNA extraction, validation of the test machine and utilizing trained operators. It is also important to remember that each sample site (nasopharynx, oropharynx sputum, endotracheal aspirates, bronchoalveolar lavage) needs to be validated.

**Host factors:** Viral shedding varies at different anatomic sites. For example, the virus was most reliably detected in sputum followed by nasal swabs during the first 14 days of symptoms, whereas throat swabs have been found to be unreliable 8 days after symptom onset [21]. Several factors can result in failure to detect the viral infection by RT-PCR—suboptimal collection procedure, if the patient has low a viral load either due to sample collection during an early stage of infection or late in the course of infection. In such a situation, a combination strategy including RT-PCR and serology could be utilized. One study reported use of combination of RT-PCR and an IgM serologic test to diagnose COVID-19 and noted RT-PCR positivity rates were >90 percent on days 1 to 3 of illness, <80 percent at day 6, and <50 percent after day 14 [22].

Higher viral loads have been noted in patients with severe illness and there is a higher chance of a false negative test with mild illness. The severity of illness may also affect the duration of viral shedding. Yang et al. reported 76 patients with different levels of illness severity, the mean viral load of severe cases was 60 times higher than that of mild cases. Mild cases were found to have early viral clearance, with 90% of these patients repeatedly testing negative on RT-PCR by day 10 post-onset. By contrast, all severe cases still tested positive at or beyond day 10 post-onset [23]. Additionally, prolonged viral RNA detection does not necessarily indicate ongoing transmissibility as the chance of detecting viable virus, with the ability to replicate, decreases after onset of symptoms reported by Wölfel et al. [24].

Given our evolving understanding of the disease process, there is a possibility of intermittent or prolonged viral shedding as noted in a few cases where the patient retested positive after a negative test [25]. Replication-competent virus was not detected in specimens from patients who recovered from initial SARS-CoV-2 infection and subsequently developed new symptoms with a repeat positive test by RT-PCR [26,27]. Repeating NAAT in a recovered patient is usually not recommended for at least 90 days unless there is clear concern for repeat infection with symptoms consistent with SARS-CoV-2 infection and without any alternative explanation [28]. In this scenario, a positive test does not necessarily indicate reinfection as prolonged respiratory shedding of viral RNA following acute infection can be observed. However, due to waning immunity from infection and the possibility of genetic drift, the chances of SARS-CoV-2 reinfection is expected to increase with time after recovery from initial infection, especially with new evolving variants as initially reported from South Africa, UK and Brazil but now spreading worldwide [29]. These variants have been seen to spread more easily and quickly.

There have been a few cases reported to have positive chest CT findings with initial negative results RT-PCR, but subsequent testing was positive [30,31]. This information has led to the questioning of sensitivity of PCR. RT-PCR may have been falsely negative due to improper sample collection with resultant insufficient viral material in the specimen or due to variability in the rate of viral detection from different tests. In addition, the viral load could have been below the limit of detection at the time of sample collection and due to different sample collection sites such as throat specimens (collected in majority of the patients in this report), has been reported to carry lower viral loads compared to nasopharyngeal specimens.

NAATs are highly sensitive and a negative test in a patient (if the specimen is obtained appropriately at appropriate timing of illness and processed appropriately) would indicate that a person is either not infected or may have been infected in the past and already recovered from the infection. It is also important to understand that positive results do not rule out bacterial infection or co-infection with other viruses.

**Serology tests:** Antibodies are detected against two major antigenic targets of SARS-CoV-2 virus: spike glycoprotein (S) and nucleocapsid phosphoprotein (N). S protein is present on the surface of the virus and plays an important role in entry. N protein interacts with the viral RNA. There are two types of antibodies—binding (binds to virus, alerts immune cells about its presence but does not interfere with its infectivity) and neutralizing (bind to virus, prevents interaction with host cell, thus rendering it non-infectious, and neutralizes effects of virus without need for host immune cells for its function) [32].

Binding antibody tests can detect IgG, IgM, and IgA by using purified proteins of SARS-CoV-2 instead of live virus. IgM is most useful for determining recent infection as it usually becomes undetectable weeks to months following acute infection, while IgG may remain detectable for months. Studying the decline in SARS-CoV-2 antibodies after mild infection among frontline health care workers, there was higher likelihood of antibody detection at 60 days post initial antibody testing in the participants with higher initial antibody response [33]. In a recent study from Japan, a stronger antibody response was noted in patients with greater disease severity. These observations support that duration of IgG antibody detection depends on the severity of the infection and the height of the initial antibody response [34]. The significance of IgA is yet to be determined in this type of infection and generally plays a role in mucosal immunity. The Infectious Diseases Society of America (IDSA) recommends against using IgA to detect evidence of past SARS-CoV-2 infection and makes no recommendation either for or against using IgM antibodies to detect evidence infection [35]. Hence, IgG is the most commonly tested antibody.

POC tests can be performed on blood samples obtained by fingerstick rather than venipuncture. They detect IgG, IgG and IgM, or total antibody. They are single-use, low-throughput tests where the presence of antibody is demonstrated by a color change on a paper strip. However, at this time, these POC serologic tests carry the risk of high false positive results. CDC’s serologic test has a sensitivity of 96% and specificity of greater than 99% based on performance evaluations [36].

Neutralization antibody tests determine the functional ability of antibodies to prevent infection of virus in vitro using a plaque-reduction neutralization test (PRNT). The PRNT utilizes the ability of antibody in serum to neutralize virus, in turn preventing the virus from causing the formation of plaques in a cell monolayer. This assay involves mixing a constant amount of virus with dilutions of the serum specimen being tested, followed by plating of the mixture onto cells of an appropriate cell line for SARS-CoV-2 virus. The concentration of plaque forming units can be determined by counting the number of plaques formed. The number of plaques in an individual well is divided by the original number of virions to calculate the percentage neutralization. Interpretation is typically based on 70% neutralization, which is the last dilution of serum capable of inhibiting 70% of the total plaques. These tests may take up to 5 days to complete. This process relies on culturing live virus and hence requires an appropriate biosafety level (BSL-3) laboratory setting.

## 4. Factors Influencing Interpretation of Results

Some individuals may not develop detectable antibodies after infection while, in others, it is possible that antibody levels could wane over time to undetectable levels. Antibodies may not be present among those tested early in illness. The presence of antibodies may also reflect previous infection and may be unrelated to the current illness. As a result, serologic test results cannot be used as definitive evidence of the presence or absence of current or previous infection with SARS-CoV-2. The IDSA, however, does have a weak recommendation that IgG antibody could be used as evidence of COVID-19 infection in symptomatic individuals if there is a high clinical suspicion but NAAT test is repeatedly negative. Until further data are available, the CDC recommends that antibody results should neither be used to make decisions about return to work, changing work or personal protective equipment requirements for health care workers nor used to determine rooming arrangements in group settings such as prisons or dormitories [32]. However, it can be certainly helpful in identifying donors with high titers for FDA-approved therapeutic plasma. The titration of neutralizing antibodies would also enhance our understanding for vaccine research. Of note, titers for antibodies are higher than neutralizing ones due to higher sensitivity of bindings assays in general. Serologic testing is also utilized to support multisystem inflammatory syndrome diagnosis in children.

Some of the antibodies are protective/neutralizing but the duration of this protective effect in not known at this time. In addition, the correlation of binding antibody titers to neutralizing titer and the ability of antibodies to protect from repeat infection are yet to be determined. Although animal challenge studies demonstrate protection in the short term, demonstration of long-term protection in humans will require future study. Hence, pending additional data, the presence of antibodies cannot be equated with an individual’s immunity from SARS-CoV-2 infection. Although tests to detect neutralizing antibodies have been approved by FDA under EUA and would be helpful to study the response to infection or vaccination, they may not have a diagnostic advantage over binding antibody tests since correlates of protection have not yet been established. In addition, T-cells and cytokines likely play a role in clearance of viral infection as well as varied titers of neutralizing antibodies [37,38]. If a serologic test to identify prior infection is performed in an individual who has received a spike protein COVID-19 vaccine, a test that detects antibodies to antigens other than the spike protein should be used.

## 5. Conclusions

Viral tests (molecular or rapid antigen tests) should be used to diagnose acute SARS-CoV-2 infection, but a negative result does not rule out infection if there is high clinical suspicion, as several factors like variability in the sensitivity of the different assays and the stage of the disease can affect the results. Molecular and rapid antigen tests have high specificity compared to antibody testing. Antibody testing should not be used to diagnose or exclude SARS-CoV-2 infections or to inform infection status as negative results do not rule out SARS-CoV-2 infections, particularly for those individuals who have been exposed to the virus and are still within the incubation period. In other words, false-negative results are more typical for samples collected too early (mostly true for serological assays) and too late (in case of PCR-based assays). As more tests are being developed with the goal for rapid results without compromising the sensitivity and specificity, the clinicians should continue to expand their knowledge on these rapidly evolving tests for timely and accurate decision making for patients.
